# Performance of Mobile LiDAR in Real Road Driving Conditions

**DOI:** 10.3390/s21227461

**Published:** 2021-11-10

**Authors:** Jisoo Kim, Bum-jin Park, Chang-gyun Roh, Youngmin Kim

**Affiliations:** Department of Highway & Transportation Research, Korea Institute of Civil Engineering and Building Technology, Goyang 10223, Gyeonggi-do, Korea; js.kim0331@kict.re.kr (J.K.); rohcg@kict.re.kr (C.-g.R.); ymkim1007@kict.re.kr (Y.K.)

**Keywords:** automated vehicle, LiDAR, real road, performance, empirical test, point cloud, intensity

## Abstract

The performance of LiDAR sensors deteriorates under adverse weather conditions such as rainfall. However, few studies have empirically analyzed this phenomenon. Hence, we investigated differences in sensor data due to environmental changes (distance from objects (road signs), object material, vehicle (sensor) speed, and amount of rainfall) during LiDAR sensing of road facilities. The indicators used to verify the performance of LiDAR were numbers of point cloud (NPC) and intensity. Differences in the indicators were tested through a two-way ANOVA. First, both NPC and intensity increased with decreasing distance. Second, despite some exceptions, changes in speed did not affect the indicators. Third, the values of NPC do not differ depending on the materials and the intensity of each material followed the order aluminum > steel > plastic > wood, although exceptions were found. Fourth, with an increase in rainfall, both indicators decreased for all materials; specifically, under rainfall of 40 mm/h or more, a substantial reduction was observed. These results demonstrate that LiDAR must overcome the challenges posed by inclement weather to be applicable in the production of road facilities that improve the effectiveness of autonomous driving sensors.

## 1. Introduction

Around the world, technological advancements are being made to expedite the commercialization of automated vehicles. Standalone automated vehicles require commercial technologies for sensors, perception and detection, and control [1]. The sensor aspect involves technologies for collecting information about surrounding conditions and objects; the perception and detection aspect involves technologies for classifying and tracking objects based on the collected data; and the control part involves technologies for controlling a vehicle based on the information about the classified object. In addition to these technologies, there is a pressing need for the commercialization of technologies for communication, precision maps, and roads and road infrastructure for connected and automated vehicles [2].

In automated vehicles, cameras, radar, and mobile light detection and ranging (LiDAR) are the most commonly utilized sensors, which act as the eyes of drivers [3]. LiDAR, which has advanced rapidly in recent years, has positive characteristics such as excellent object detection, high detection accuracy, and high performance even under low-light conditions [4]. Because of these advantages, LiDAR is the most suitable sensor for automated vehicles and is being actively used by automakers such as Google Waymo and Volvo. It is no exaggeration to say that LiDAR has led to the recent rapid development of automated vehicles [5]. However, LiDAR has a few shortcomings. The performance of LiDAR systems, which are equipped with a laser scanning system, may differ depending on the location and the reflectivity of the object to be sensed by the systems [6]. On rainy days, the scattering of the laser caused by raindrops interferes with the object detection, with errors having been noted as the measurement distance to an object increases [7].

The positive or negative characteristics of LiDAR should be identified with a performance verification performed in a real road environment. However, only a few studies have verified and highlighted the performance of LiDAR through empirical tests in real road environments. LiDAR was initially developed for collecting land information in the field of aerial surveying and to develop the Geographic Information System (GIS). However, its use in automated vehicles is relatively recent [8], and the disclosure of performance is perceived as revealing the technological secrets of a company [9]. It is also argued that the government should take the initiative in establishing a performance verification system for the safety of automated vehicles because of accidents that have occurred with automated vehicles using LiDAR, such as in the case of the Uber self-driving car [2].

Considering the current situation concerning the urgent commercialization of automated vehicles and the associated safety issues, the qualitative characteristics of LiDAR mentioned in the literature should be promptly verified in real-road driving. Furthermore, the results of such performance verification should be quantified using the appropriate performance indicators.

This study started from the question whether the LiDAR characteristics known mostly from lab- or simulation-based studies are the same in real-road driving environment. In order to identify this problem, this study explores the performance of LiDAR systems when driving in a real-road environment by selecting performance indicators that can be quantified for objectifying the results of performance verification. The performance of LiDAR is verified by observing the change in performance indicator values through scenario-based experiments. we conducted a performance evaluation while changing the road environment at the site. When performing the test, a real road environment was created by utilizing a meteorological environment demonstration facility (Yeoncheon SOC Demonstration Research Center of the Korea Institute of Civil Engineering and Building Technology (KICT)), and the materials used in real traffic signs in Korea and representative materials frequently encountered while driving were used as the objects for detection. The data were collected using the LiDAR system, which was mounted on an automated vehicle being tested by the KICT.

**Figure 1:**
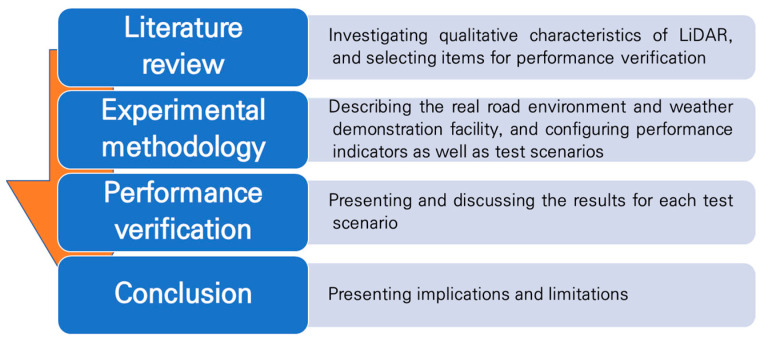
Structure of the study.

This paper is organized into four stages as shown in Figure 1. Firstly, the qualitative characteristics of LiDAR are summarized through a literature review. From these, the characteristics that need to be verified with the selected quantitative indicators while driving an automated vehicle in a real road environment are sorted out. Subsequently, the experimental methodology, including the real road environment, items for performance verification, performance indicators, and scenarios are presented. The results of the performance verification conducted according to the experimental methodology are revealed, and the performance of the LiDAR is examined for each test item. Finally, the suitability of LiDAR for use in automated vehicles in terms of performance is discussed, and the implications and limitations are described.

## 2. Literature Review

Global Navigation Satellite System (GNSS) technologies have been widely used commercially since the early 1990s. Hence, LiDAR technologies have become well-established surveying techniques for acquiring geospatial information [8]. Miniaturized mobile LiDAR (in this study, LiDAR refers to the mobile LiDAR used for automated vehicles or traffic information collection) is almost the same technology as airborne or terrestrial LiDAR, but it has undergone rapid advancement over a short period of time. LiDAR is used to collect various traffic-related data, such as speed, road, and road facility information, and has the advantage of being more accurate than other methods of collecting traffic information [10]. LiDAR radiates a laser with a wavelength in the range of 760–1900 nm in the near-infrared band and detects objects using the phase-shift and time-of-flight (TOF) methods. The TOF method, which has a high irradiation speed with little effect on the distance to an object, is commonly used [11].

Automated vehicles use LiDAR for object detection, perception, and localization. The data collected by LiDAR are used in automated vehicles for object detection, categorization, tracking, and motion prediction [5]. Automated vehicles rely on LiDAR to obtain information about dynamic objects such as pedestrians, traffic lights, and surrounding vehicles to ensure safety [4]. Google’s Waymo, regarded as the best automated vehicle in existence, also uses LiDAR to detect unexpected objects [9]. The intensity of LiDAR is collected to create an intensity map of the area for driving guidance [12]. As such, LiDAR is at the forefront of the current rapid development of automated vehicles, and automated vehicles use it to gather a range of information required for safe driving [5].

The performance of LiDAR is determined in terms of eye safety, accuracy, field-of-view (FOV), data rate, scan frequency, and range [11]. The waveform of the laser used in LiDAR affects the accuracy and precision of the measurement [13]. Such performance is indicated in the product performance manual provided with the purchase of the LiDAR. However, the realization of the specified performance when driving the automated vehicle is not guaranteed. Theoretically, the higher the frequency, range, and FOV, the more information can be scanned, making it easier to acquire information on the real road [14].

High-spec LiDAR products with more than 64 channels are commonly used in automated vehicles. Theoretically, the driving speed of automated vehicles has no effect on the object detection performance of LiDAR [2]. It becomes easier to perform the measurement of the distance to an object using the LiDAR, with the reduction in distance between the object and the automated vehicle [2]. Because LiDAR utilizes the reflection of laser pulses from the object, its performance is affected by the material and the color of the reflective surface of the object. The performance of LiDAR is better when the color of the object is white as opposed to a darker achromatic shade and when the material has high reflectivity [6]. The performance of LiDAR is an important factor when manufacturing a LiDAR-based automated vehicle to be operated in a real road environment. Nevertheless, the mentioned performance of LiDAR is only based on simulation, lab tests, or theoretical reasoning, and studies on the performance verification of LiDAR on the real road are difficult to find. This is mainly because the history of LiDAR technology is rather brief. In addition, as automated driving technology has not yet been commercialized, companies are reluctant to reveal the performance of their automated vehicles, as it may indicate their level of automated vehicle technology [9].

The problems associated with LiDAR, such as the high production costs and the short durability of the motor caused by the rotation of LiDAR, still hinder its commercialization. Recently, the utilization of non-rotating, solid LiDAR has increased [3], and the use of LiDAR in combination with image sensors has also increased [15]. In particular, the fusion of image sensors and LiDAR exhibits superior performance when compared to that of an image sensor alone [16]. For this reason, the development of fusion technology with multiple sensors is being actively pursued to improve the driving safety of automated vehicles [17]. In some studies, sensor fusion technology has been verified to be effective in real road environments under various weather conditions (clear/cloudy/rain/weak snow), considering the performance of LiDAR sensors. However, there has been no discussion about how much LiDAR data increased or decreased while controlling various environments such as ‘changes in the speed of LiDAR’ and ‘changes in the materials.’ The amount of rainfall was not varied or controlled, and experiments were performed only at close distances (1.5, 3.0, 4.5 m) [18].

Many studies have mentioned the degradation of LiDAR performance, arguing that the performance of automated vehicles in rain, fog, and snow requires further development to ensure safety [19]. The performance degradation of LiDAR in rain is explained by the following two reasons: Firstly, the number of point clouds (NPCs) obtained by the reflection of the laser off an object is reduced significantly due to collisions with raindrops on rainy days. Secondly, the accuracy of the distance to the object is degraded as the laser returns after colliding with raindrops instead of the object. It has also been suggested through simulation that the maximum range, NPCs, and the obstacle detection range of LiDAR decreases due to rainfall [7]. Furthermore, it has been verified that rainy weather can significantly affect detection accuracy through tests conducted to detect pedestrians using LiDAR mounted on automated vehicles in real parking lots [4]. In addition to rain, fog and snow also degrade the performance of LiDAR for the same reasons [20]. The performance of LiDAR in the perception of objects (vehicles and pedestrians) is reduced under foggy and rainy weather when using real-world data, in terms of indicators such as NPC, intensity, and echo pulse width (EPW). A new object perception algorithm has been developed to overcome LiDAR performance degradation due to harsh weather environments [21]; however, data changes in various environments such as the amount of rainfall, distance/speed change, and material were not presented. Data such as intensity, EPW, and NPCs of atmospheric layer and asphalt were collected in another study using LiDAR for predicting clear, rainy, foggy, and snowy conditions [22]; however, data changes with regard to various environmental factors such as the amount of rainfall, distance/speed change, and material were not considered. In a survey of experts in the field of the production of automated vehicles, the weather-related issue was pointed out to be a matter that needs to be addressed promptly [23].

Despite its active utilization in automated vehicles, there are few studies which have verified the performance of LiDAR in real road environments. However, considering that LiDAR currently plays a key role in object perception, tracking in automated vehicles, performing an empirical test to verify the performance of LiDAR with respect to the aforesaid characteristics seems to be essential at the present time when looking at the commercialization of automated driving. In addition, there is an urgent need to determine the qualitative characteristics of LiDAR for testing and to select the characteristics or test items that require performance verification in real road environments. The test items for performance verification of LiDAR in this study, which were chosen from among various performance characteristics of LiDAR presented in the literature or product description, are summarized in Table 1. These test items were chosen as they can be quantified and are essential for the safe driving of real automated vehicles. Hereafter, the performance of the LiDAR system refers to the object-detection performance.

As suggested in several studies, the item for performance verification, the “object detection performance according to weather conditions (rainfall)”, is known to show degradation in performance on rainy days. However, this study attempted to observe the effect of the degradation in performance on the intensity value, a performance indicator (refer to the performance indicator of LiDAR in the next chapter), using a rainfall demonstration facility in a real road environment. Furthermore, the degradation of the object detection performance of LiDAR with an increase in the amount of rainfall could be examined by artificially adjusting the amount of rainfall. The repeated test results for performance verification in real road environments are expected to justify the use of LiDAR, and it is expected to enable comprehensive assessment of the performance of LiDAR considering the effects of the road environment and weather.

## 3. Methodology of Testing

### 3.1. Purpose of the Test, Items for Performance Verification, and Performance Indicators

#### 3.1.1. Purpose of the Test and Items for Performance Verification

The purpose of the test is to evaluate the items for performance verification listed in Table 1 based on the performance indicators in a real road driving environment. In other words, it aims to examine how the qualitative characteristics of LiDAR identified through literature are reflected in the real road driving environment by analyzing the performance of LiDAR based on quantitative performance indicators.

In this study, the detection performance of LiDAR is evaluated with respect to the following parameters: the driving speed of the automated vehicle (performance by speed), distance between the target object and the LiDAR (performance by distance), material of the target object (performance by material), and changes in weather conditions, especially rainfall (performance by rainfall). These test items were examined based on two performance indicators as follows.

#### 3.1.2. Performance Indicators for LiDAR

The performance indicators that verify the performance of LiDAR are the NPCs and the intensity of LiDAR.

NPC refers to the number of laser beams that are radiated from LiDAR reflected from the target object and returned to the LiDAR receiver. An object can be perceived using LiDAR data in a situation where point clouds are acquired over a certain scale that can be clustered. In similar study, the authors discovered changes in the max range, number of points in scan (identical to NPC), and obstacle detection range of LiDAR with increasing rain rate through a simulation [7]. The number of hit points (identical to NPC) is used to compare with different weather conditions in another study [18]. NPC is judged the most suitable indicator to describe visibility, among these three indicators. This is because under the same conditions, the larger the NPC value, the more precisely the shape of the object can be expressed. The reasons for excluding max range and obstacle detection range are as follows: Since max range means a case where even one point is collected, it was judged that it is not adequate to describe the performance of LiDAR. Similarly, the obstacle detection range can be changed depending on the perception algorithms. Therefore, the more NPCs, the more advantageous it is to cluster the point cloud to accurately identify the shape of the target object.

Intensity refers to the intensity of the reflected and returned laser beams compared to the intensity of the laser beams radiated from the LiDAR. In general, intensity is expressed as a number between 0 and 1, where 0 indicates that no light is transmitted from the laser diode, and 1 indicates that the entire emitted laser is reflected. It may be affected by factors including the irradiation angle and range of the LiDAR, as well as surface material, color, roughness, and humidity of the irradiated object. As it does not represent a constant value due to various variables, only a relative comparative measurement value rather than an absolute value is obtained [2].

**Figure 2:**
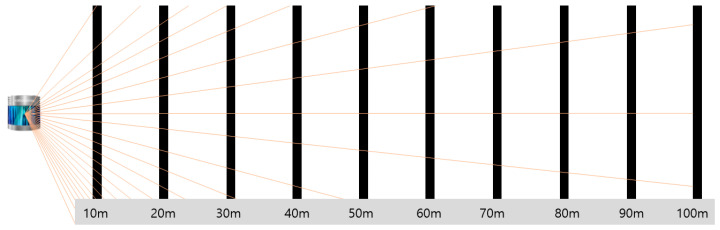
Side view of laser beams arriving at each distance.

The performance indicators for LiDAR are based on the distance between the target object and the LiDAR. This is because the number of laser beams reflected by the object increases as the distance between the LiDAR and the object decreases with the rotation of the LiDAR used in the test. As shown in Figure 2, when the distance between the target object (black bars in the figure) and LiDAR is 10 m, the number of laser beams inevitably becomes larger than when the distance is 100 m. Therefore, the performance of the LiDAR should be compared at the same distance.

The LiDAR used in this study is RS-LiDAR-32, manufactured by Robosense, and its technical specifications are shown in Table 2.

#### 3.1.3. Performance Indicator Verification Method

The performance of LiDAR was verified by the following procedures.

Firstly, a comprehensive analysis was performed using graphs of the NPC and intensity based on the data acquired through the test. The trends of the NPC and intensity values were observed while interpreting the graphs.

Secondly, statistical analysis was performed to determine whether there was a difference between the groups for each item in terms of distance/speed/material. As shown in Figure 2, there seemed to be a difference between distance groups among the items according to the distance, considering the working principle of LiDAR. Therefore, statistical analysis was performed while maintaining the same distance.

The statistical analysis method and the results of this study are as follows:

Analysis of variance (ANOVA) was performed to identify whether there was a difference in NPC or intensity depending on changes in the target material at the same vehicle speed at each distance (20, 40, 60, 80, and 100 m), or whether there was a difference in NPC or intensity depending on changes in speed for the same material at each distance (20, 40, 60, 80, and 100 m).

ANOVA is a method of testing whether the difference between the means of samples obtained from several populations is a statistically significant difference using the F-distribution. When comparing the within-variance (F-rejection value) and the between-variance (F-ratio) representing each population, if the between-variance is sufficiently larger than the within-variance, it is judged that there is a difference in the mean for each population [24]. Therefore, this study intends to analyze whether differences occur in NPC and intensity according to changes in material groups and speed groups for each distance by using two-way ANOVA.

The ANOVA results can be explained based on the following example.

If the same speed group does not show statistical differences in NPC according to changes in the material at a distance of 20 m on a sunny day, our hypothesis is considered valid, and the same speed group (e.g., 80 km/h) for each material does not affect the observation of NPC and performance of LiDAR.

If the same material group does show statistical differences in NPC according to changes in speed, our hypothesis is rejected, and the same material group (e.g., aluminum) for each speed does affect the observation of NPC.

For the first and second verification procedures mentioned above, the performance items were evaluated under sunny (rainfall of 0 mm) and rainy (10, 20, 30, 40, and 50 mm) conditions.

### 3.2. Configuration of the Test Environment and Test Scenarios

#### 3.2.1. Configuration of Test Environment

**Figure 3:**
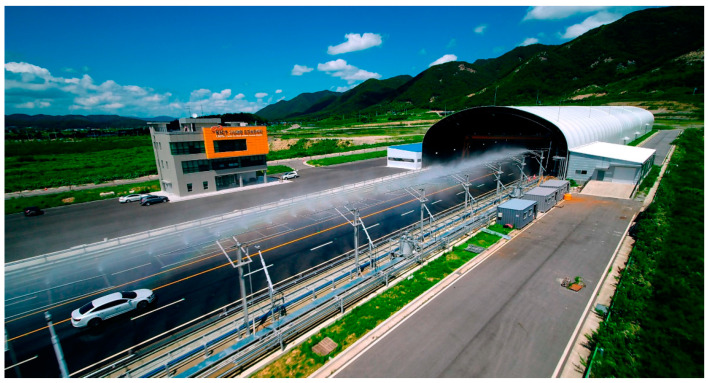
Test site (KICT SOC Demonstration Research Center).

For verification under conditions similar to real road environments, the test was conducted on a test road equipped with meteorological environment demonstration facilities at the Yeoncheon SOC Demonstration Research Center of the KICT (Figure 3). A similar test using a vision sensor (Mobileye) was performed at the same site [25]. The site consists of a multi-lane section paved with asphalt, as shown in the figure. As there was a straight road section of 600 m or longer, the data could be acquired by reaching the target speed with sufficient acceleration. On the right side of the lane, there is meteorological demonstration equipment for reproducing rainfall conditions of 50 mm/h or more, allowing smooth LiDAR data acquisition according to changes in speed/distance/rainfall. The test was performed from 10:00 to 17:00 for 3 days, and the temperature was between 13 and 20 °C during the test.

**Figure 4:**
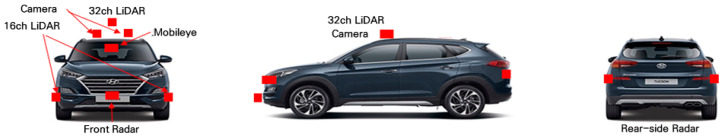
Configuration of the data gathering facility (automated vehicle).

The vehicle that acquired the data was an automated vehicle manufactured by the Korea Institute of Construction Technology equipped with a LiDAR, a radar, and vision sensors (Mobileye and cameras, which were not used in the study), as shown in Figure 4. As this study aimed to verify the object detection performance of the LiDAR, data were acquired and analyzed only with the 32ch LiDAR installed on the roof of the vehicle.

**Figure 5:**
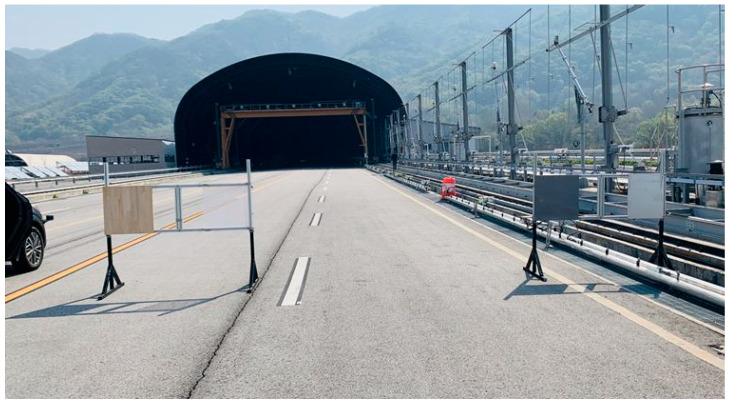
Object targets.

The object target, which is the target for data acquisition, was manufactured in the shape of a 60 cm × 60 cm square with reference to the specifications of road traffic signs. As shown in Figure 5, two targets were installed at a height of approximately 1.0 m on the frames on the left and right sides of the driving path of the vehicle. Therefore, the data for the four targets were acquired simultaneously during a test.

**Figure 6:**
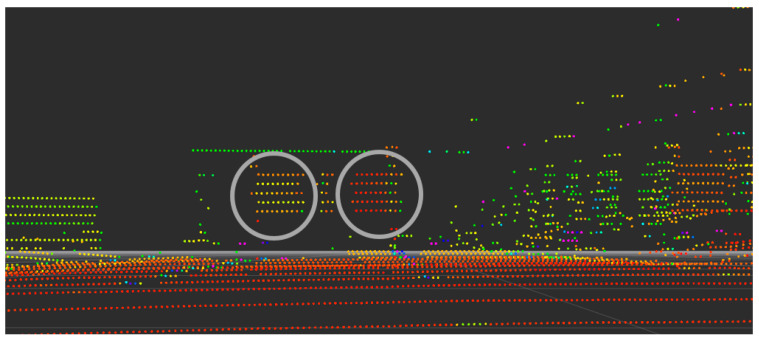
Object target displayed in point clouds (within gray circles).

The object targets (Figure 5) detected by LiDAR and expressed as a point cloud are shown in Figure 6. In the close situation, as shown in the figure, the frame to which the target is attached is also detected. Additionally, the two targets are clearly distinguished, and the NPC and intensity are analyzed for the point cloud included in this area.

#### 3.2.2. Test Scenarios

The performance verification items were divided into environmental factors and target factors, and the various test scenarios are shown in Table 3. The scenarios by test item for performance verification in Table 3 were subdivided according to speed/distance/rainfall/material in various ways. These scenarios were then combined and tested repeatedly.

However, the actual test was performed in a complex manner to ensure the efficiency of the experiment.

Speed refers to the moving speed of LiDAR mounted on automated vehicles. The test was conducted at four different speeds ranging from 20 km/h to 80 km/h, at intervals of 20 km/h, and repeated five times. Distance refers to the distance between the LiDAR and the target. By examining the location coordinates for each distance from the target in advance, the data could be automatically acquired while driving at each speed. Therefore, assuming that the vehicle was driven once at a speed of 80 km/h and under a condition of 10 mm/h rain on a sunny day, the following data items were collected. In the file for one drive, the performance index data for four different materials were collected at a distance of 20, 40, 60, 80, and 100 m.

Rainfall was reproduced in a range from 0 mm/h (sunny day) to 50 mm/h at 10 mm/h intervals. To minimize the influence of wind when demonstrating rainfall, the anemometer was checked to ensure that the vehicle is driven only in situations not affected by wind (5 m/s or less).

As for the target, wood, plastic, steel, and aluminum were selected for comparison by referring to vehicles, trees, and signs frequently encountered on the roadside during real road driving.

## 4. Results and Discussion

### 4.1. Performance Index and NPCs

#### 4.1.1. Sunny Day

**Figure 7:**
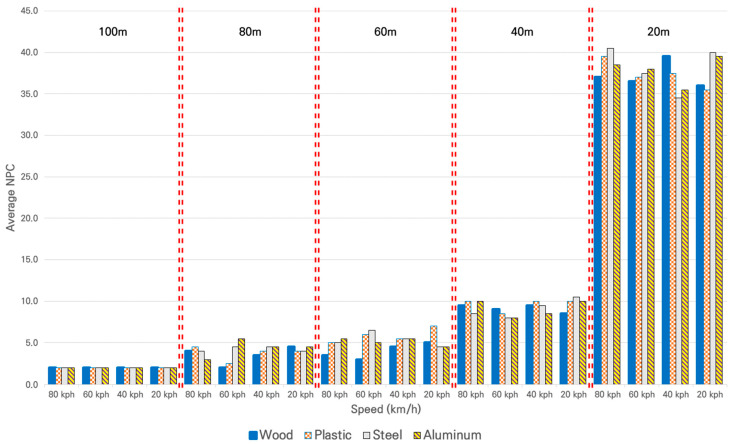
Average of NPC for different materials at each distance and speed on a sunny day.

The data acquired on a sunny day are shown in Figure 7. In the figure, the *X*-axis is configured to observe the distance and speed together, and the *Y*-axis indicates the average value of the NPC.

The trend of the NPC values according to distance has the following characteristics: As shown in Figure 7, at the same distance, the NPC exhibited a constant value. For all the materials, the NPC increased as the distance decreased. Furthermore, the NPC increased as the distance decreased at all the speeds. In particular, it gradually increased from 100 m to 60 m and then rapidly increased from 40 m to 20 m. This explains the behavior shown in Figure 2.

Secondly, the statistical differences in the NPC collected at each distance on a sunny day with the changes in material or speed were examined by ANOVA. Therefore, the changes in the NPC with the changes in material within the same speed group were analyzed by setting Hypothesis 1 of the ANOVA as “There is no difference in NPC depending on the type of material”. The changes in the NPC according to the changes in speed within the same material group were analyzed by setting Hypothesis 2 of the ANOVA as “There is no difference in NPC depending on the speed”. Table 4 shows the results of ANOVA. Table 4 presents whether each hypothesis was accepted/rejected without showing the details of ANOVA, such as between-variance and within-variance, due to the extensive amount of data.

At 100 m, ANOVA could not be performed as all measured values were the same, which could be interpreted as the absence of any difference according to material or speed. Hypothesis 1 was accepted for all other distances. In other words, there was no statistical difference in NPC according to changes in material in the same speed group. Hypothesis 2 was accepted for all distances except for 100 m. In other words, there is no statistical difference in NPC according to changes in speed in the same material group.

The results of the comprehensive analysis verifying the performance of LiDAR based on NPC as the performance indicator on a sunny day were as follows. It was analyzed that performance on a sunny day was unaffected by distance, speed, or material, based on which the following results could be inferred regarding the performance of LiDAR. On a sunny day, LiDAR performs better at a closer distance to the target, and the movement speed of LiDAR does not cause a difference in its performance. There was no difference in the performance of the LiDAR caused by any material used in this experiment. Rather than interpreting this as “LiDAR could not classify objects by material,” it seemed more reasonable to interpret it as “the LiDAR performance indicator NPC used in this analysis was not suitable for classifying objects in terms of the target material”.

#### 4.1.2. Rainy Day

Table 5 shows the amount of rainfall per hour prior to describing the experimental results in a rainfall environment. The table suggests that the driver’s eyes started to be affected by rain when the rainfall was more than 20 mm.

**Figure 8:**
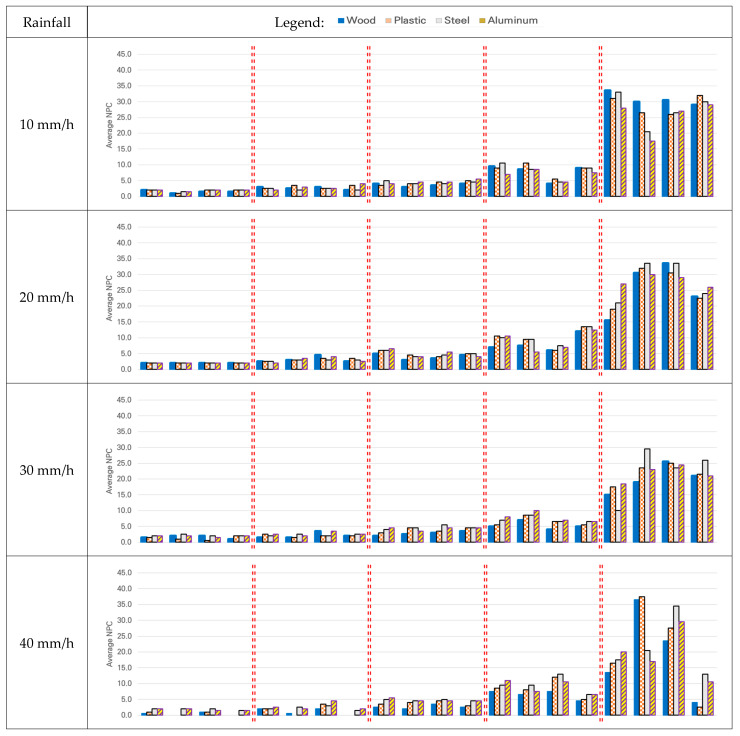

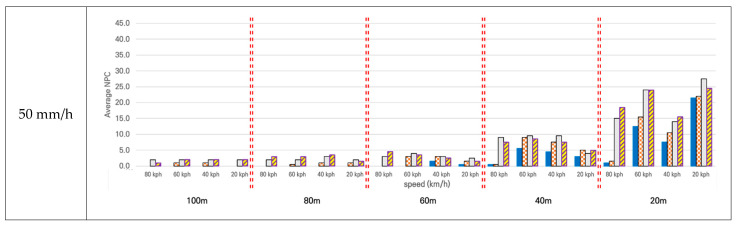
Average NPC at each distance for different amounts of rainfall.

Figure 8 shows the average NPC for each distance for different categories of rainfall.

The NPC by distance increased as the vehicle approached the target, and this was consistent for all materials. Moreover, this was the same as the result obtained on a sunny day. However, the NPC by distance decreased as the rainfall increased. For steel and aluminum, having relatively high reflectivities, data could be collected for each distance without operating the wipers, even at a rainfall of 50 mm/h when the targets could not be identified with the naked eye (see Table 5), but the NPC was lower compared to that on a sunny day. For wood and plastic, data collection at a distance of over 80 m during a rainfall of 40 mm/h and over 60 m during a rainfall of 50 mm/h was reduced compared to that on a sunny day. In other words, compared to a sunny day, the decrease in NPC was greater for wood and plastic than for steel and aluminum. For example, at a distance of 60 m during a rainfall of 50 mm/h, the ratio of reduction in NPC compared to that on a sunny day for each of these materials was: 87.5% for wood, 68.1% for plastic, 41.9% for steel, and 41.5% for aluminum. The most extreme NPC reduction compared to the sunny day was measured under rainfall of 50 mm/h as follows: by up to 100% for wood when perceiving the target at a distance of 100 m; by up to 83.3% for plastic when perceiving the target at a distance of 80 m; by up to 45.7% for steel when perceiving the target at a distance of 20 m; and by up to 45.7% for aluminum when perceiving the target at a distance of 20 m.

Secondly, the statistical differences in the NPC collected at each distance on a rainy day according to the changes in material or speed were examined by ANOVA. Therefore, as in the analysis for the sunny day, Hypothesis 1 of the ANOVA was set as “There is no difference in the NPC depending on the type of material” for a rainfall of 10, 20, 30, 40, and 50 mm/h. Hypothesis 2 of the ANOVA was set as “There is no difference in the NPC depending on the speed” for a rainfall of 10, 20, 30, 40, and 50 mm/h. Table 6 shows the results of ANOVA.

As the measured values were the same for Hypotheses 1 and 2 under the conditions of 100 m distance and 20 mm/h rainfall, ANOVA could not be performed, which could be interpreted as the absence of any difference according to material or speed.

For Hypothesis 1, when the rainfall was 30 mm/h or more, there was a statistical difference in NPC according to changes in material in the same speed group at all distances (except for 30 mm/h rainfall at a distance of 100 m/80 m, and for 40 mm/h rainfall at a distance of 20 m). For Hypothesis 2, when the distance was 40 m or 20 m, there was a statistical difference in NPC according to changes in speed in the same material group regardless of the amount of rainfall. Although there were some exceptions, at 100 m, 80 m, and 60 m, the hypothesis was generally accepted as that on a sunny day.

The results of the comprehensive analysis verifying the detection performance of LiDAR based on NPC performance indicators on a rainy day are as follows. The intensity decreased as the rainfall increased. This was consistent with previous research showing that the maximum range and NPC were reduced as the number of laser beams was reduced owing to the raindrops [7]. Statistical analysis revealed that the increase in rainfall, especially to 30 mm/h or higher, caused a statistical difference in the performance according to material, improving the NPC performance indicators for LiDAR compared to those on a sunny day. In the rain, the NPC acquisition performance of LiDAR improved at a close range compared to that on a sunny day. Based only on the numerical values of the performance indicator NPC, the performance of LiDAR decreased as the rainfall increased, but the performance could be improved as the performance depends on the target materials.

The NPC decreased as rainfall increased for all materials up to a rainfall of 30 mm/h. For a rainfall of above 40 mm/h, the NPC of wood and plastic was not acquired, suggesting that the detection performance of LiDAR represented by NPC was maintained up to a rainfall of 30 mm/h. For rainfall above 40 mm/h, the material could be classified, but the detection performance of LiDAR was degraded as the NPC itself was not acquired.

### 4.2. Performance Indicator: Intensity

#### 4.2.1. Sunny Day

**Figure 9:**
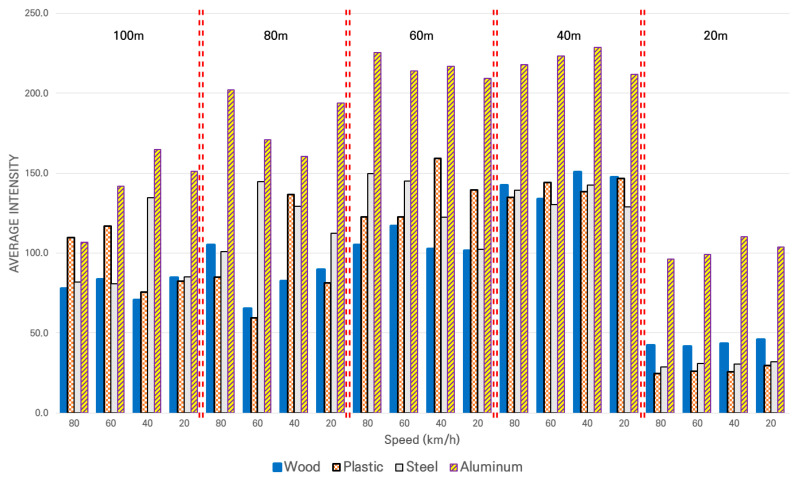
Average of intensity for different target materials at each distance and speed on a sunny day.

The data acquired on a sunny day are shown in Figure 9. The figure shows the distance and speed on the *X*-axis and the average value of the intensity on the *Y*-axis.

As shown in Figure 9, the intensity values of all materials mostly increased as the distance decreased from 100 m to 40 m. However, the intensity decreased significantly at a distance of 20 m. This may have been because the manufacturer of the LiDAR system forced the intensity to be low at close range.

The ranges of the intensity values for each material are as follows: from 41.5 to 150.7 for wood, 24.5 to 159.3 for plastic, 28.8 to 149.8 for steel, and 96.0 to 228.8 for aluminum. The intensity of the LiDAR used in the test was in the range of 0 to 255, which could be used to convert the intensity range for each material into a percentage range as follows: 0.16% to 0.59% for wood, 0.10% to 0.63% for plastic, 0.11% to 0.59% for steel, and 0.38% to 0.90% for aluminum. The intensity was the highest for aluminum when compared to the other materials.

The statistical difference in the intensity collected at each distance on a sunny day according to the changes in material or speed were examined by ANOVA. Therefore, the intensity changes according to the changes in material within the same speed group were analyzed by setting Hypothesis 1 of the ANOVA as “There is no difference in intensity depending on the type of material”. The intensity changes according to the changes in speed within the same material group were analyzed by setting Hypothesis 2 of the ANOVA as “There is no difference in the intensity depending on the speed”. Table 7 shows the results of this ANOVA test.

Hypothesis 1 was rejected for all distances. In other words, there was a statistical difference in intensity according to changes in material in the same speed group. Hypothesis 2 was accepted for all distances. In other words, there was no statistical difference in intensity according to changes in the speed in the same material group.

The results of the comprehensive analysis verifying the performance of LiDAR based on intensity as the performance indicator on a sunny day were as follows. The materials affected the collection of intensity and the performance indicator of LiDAR, but the speed did not affect the collection of intensity. As there was a difference in the intensity depending on the material, the cases of using LiDAR to perceive and classify objects [5] and to create an intensity map for driving guidance [12], mentioned in the literature review above, represented the proper use of LiDAR.

#### 4.2.2. Rainy Day

**Figure 10:**
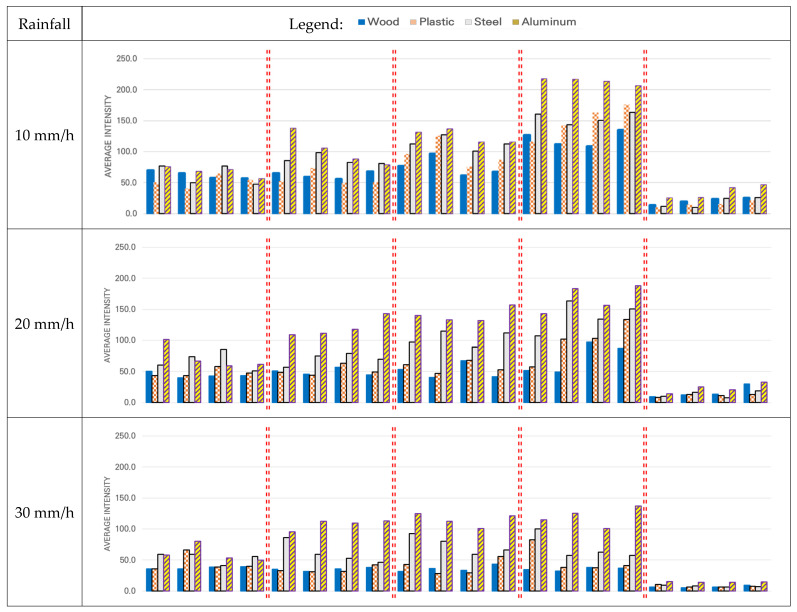

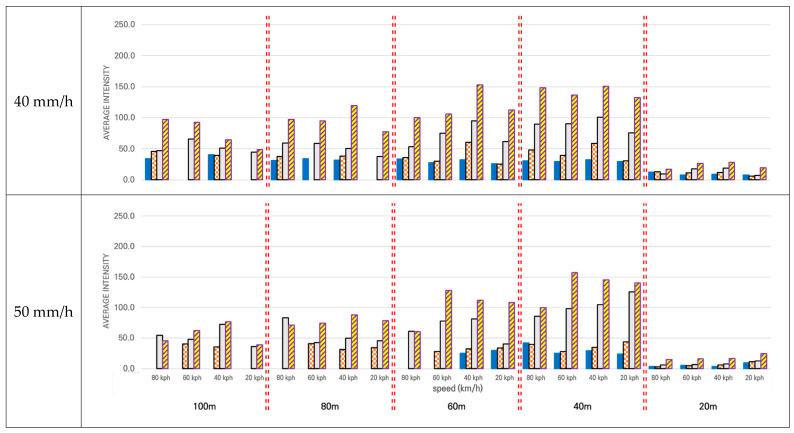
Average of intensity with speed for each distance and for each material for different amounts of rainfall.

The overall trend of intensity with distance from the target for different materials during various amounts of rainfall is shown in Figure 10.

The intensity was found to decrease as the rainfall increased. For all materials, the smallest value of intensity was observed at 20 m, which was the same as that on a sunny day. The intensity value was higher for aluminum than for the other materials in all cases. At 50 mm/h of rainfall, the intensity was not detectable for wood at 100 m and 80 m. This was because there was no intensity value as the data were not acquired (NPC = 0).

A comparison of the intensity of each material under the conditions of 50 mm/h rainfall and 60 m distance, which allowed the comparison of all four materials with those on a sunny day, showed that the intensity decreased by 74.3% for wood, 76.9% for plastic, 49.8% for steel, and 52.8% for aluminum. In addition, the ratio of maximum reduction in intensity for each material compared to a sunny day was as follows: by 87.0% for wood at a distance of 20 m during a rainfall of 50 mm/h, by 79.1% for plastic at a distance of 80 m during a rainfall of 40 mm/h, by 74.3% for steel at a distance of 20 m during a rainfall of 40 mm/h, and by 85.7% for aluminum at a distance of 20 m during a rainfall of 50 mm/h.

The statistical differences in the intensity collected at each distance during rainfall according to the changes in material or speed were examined by ANOVA. Therefore, as in the analysis for the sunny day, Hypothesis 1 of the ANOVA was set as “There is no difference in intensity depending on the type of material” for a rainfall of 10, 20, 30, 40, and 50 mm/h. Hypothesis 2 of the ANOVA was set as “There is no difference in intensity depending on the speed” for a rainfall of 10, 20, 30, 40, and 50 mm/h. Table 8 shows the results of this ANOVA test. As in the previous tables, only the acceptance/rejection of each hypothesis is presented. In Table 8, for the missing five conditions of ANOVA (100 m–40 mm/h, 100 m–50 mm/h, 80 m–40 mm/h, 80 m–50 mm/h, and 80 m–50 mm/h), the data for wood and plastic were not acquired.

For the material group of Hypothesis 1, the hypothesis was rejected at all distances regardless of the increase in rainfall, indicating a statistical difference in the intensity according to changes in material for each speed group. In the speed group of Hypothesis 2, both acceptance and rejection of the hypothesis were observed. However, in general, there was no difference in intensity depending on the speed, and the results also suggested that the change in speed indicated a difference in intensity as the rainfall increased and the distance decreased.

The results of the comprehensive analysis verifying the detection performance of LiDAR based on intensity performance indicators on a rainy day are as follows. As seen in the literature review, the performance of LiDAR was degraded by rainfall. The performance indicator intensity decreased as rainfall increased. Rather than interpreting such a decrease in intensity as “LiDAR could not be used for automated vehicles due to performance degradation during rainfall,” it seemed more reasonable to interpret it as “the performance degradation was not significant for rainfall of less than 30 to 40 mm/h, allowing sufficient utilization of LiDAR.” At the same distance, there was a statistical difference in intensity depending on the material but not in the material depending on the speed.

### 4.3. Overall Conclusions

In this study, the distance between the LiDAR of the automated vehicle and the target was divided into 20 m intervals to observe the changes in performance indicators, the NPC, and the intensity collected by LiDAR depending on changes in the speed of the automated vehicle (i.e., the speed of the LiDAR) and the material of the target. Through this, the items of performance validation for LiDAR presented in Table 1 were verified in real road environments. The far-right column of Table 9 summarizes the results of this analysis.

The verification results of LiDAR show that the NPC increased as the distance from an object decreased, and the intensity also increased. However, the manufacturer has forced the intensity value at 20 m to be measured low.

With LiDAR, the NPC was measured at a constant value regardless of the material under conditions of sunny days and rainfall of 30 mm/h or less, but it was measured as a value differentiated by material at a rainfall of 40 mm/h or more. The intensity was measured as a value differentiated by the material at the same distance, regardless of the weather.

For different speeds, NPC collection was observed to be inconsistent only at a close range of 20 m and 40 m during rainfall. This part is difficult to clearly define. In all weather conditions except this, the change in speed did not affect the NPC measurement. The intensity was measured as a constant value under all the conditions.

During rainfall, the object detection performance of LiDAR, represented by the NPC and intensity, was generally reduced. However, during a rainfall of 30 mm/h or more, the difference in NPC between the materials was detected, demonstrating enhanced LiDAR performance. The performance of LiDAR in terms of intensity was observed to not decrease significantly during a rainfall of 30 mm/h or less at a distance of 40 m or less.

## 5. Conclusions

At a time when the issues of the urgent commercialization and the safety of automated vehicles are being raised, this study aimed to verify the differences between the qualitative characteristics of LiDAR found in the literature and the laboratory measurement results in real road environments.

This paper is based on the project to develop new road facilities, such as road signs or traffic cones, that support automated driving safety. The starting point of the question in this paper is whether known LiDAR characteristics (Table 1) are same even when tested on real signs on the road. In order to identify the problem, we conducted a performance evaluation while changing the road environment at the site. Changes (or controls) of the road environment which we were interested in in our research were ① the distance between the vehicle (LiDAR) and target object, ② materials of target object, ③ the speed of vehicle (LiDAR), and ④ the amount of rainfall on the real road. The indicators used to verify the performance of LiDAR were NPC and intensity, and differences in data were identified through an ANOVA test. The experiment was performed according to a scenario composed of these four changes, and the verification results are as follows.

The NPC was found to decrease on a rainy day compared to a sunny day, and a rainfall of 40 mm/h affected the performance of LiDAR represented by NPC, as the data for some materials could not be collected. On a sunny day, there was no difference in the distance groups according to changes in material and speed. Nevertheless, with an increase in the amount of rainfall, the NPC for each type of material was measured to be different. Statistically, when the rainfall exceeded 30 mm/h, the NPC started to be measured differently for each material. As object perception using LiDAR is possible only with sufficient NPC, it seemed appropriate to utilize information on facilities or objects within 40 m based on the LiDAR used in this study. Object perception seemed possible not only on a sunny day, but also during a rainfall of 30 mm/h, in which case the NPC acquisition performance was maintained. In terms of LiDAR intensity, there was a statistical difference based on the material group, but no difference was observed in the speed group on a sunny day. This meant that materials affected the collection of intensity, whereas speed did not. During rainfall, the material group showed the same statistical difference as on a sunny day, and the following results could be inferred.

As the rainfall increased, the intensity decreased. However, as there was a statistical difference in the intensity for the material group, object perception or material classification using intensity seemed possible regardless of weather conditions. For example, considering ordinary roads surrounded by various materials such as trees and street signs, it would be possible to classify road facilities using the intensity, which is an index classified according to the material based on the results of the intensity analysis in this study. The use of materials with large differences in reflectivity is expected to help with such object perception.

Existing studies have found that the detection performance of LiDAR deteriorates under conditions of rain, fog, and snow [4,7,18,20,21,22]. This is also the result of this study, in which the detection performance of LiDAR deteriorated under rain conditions. However, the differences between this study and previous studies are as follows: ① Whereas previous studies have discovered how weather changes effect the detection performance of LiDAR for specific figured objects, such as pedestrians, this study focused on the effect on the detection performance of LiDAR when changes (in materials, etc.) were applied to the same object. ② While most of the existing studies measured data at a short distance, within 20 m, this study measured data at a distance of 100 m to 20 m. ③ Existing studies did not have clear rainfall conditions or were limited to specific rainfalls, but this study confirmed the effect on the detection performance of LiDAR while controlling the rainfall from 10 mm/h to 50 mm/h. The biggest difference of this study from previous studies is that the detection performance of LiDAR is measured in a changing situation in which all four conditions (object materials, vehicle speeds, measurement distances, and rainfalls) are combined.

We identified five items for the performance verification of LiDAR as presented in Table 1, and four out of these five items were verified in real road environments. The results of the verification show that LiDAR, which maintained its detection performance during rainfall of up to 30 mm/h, has the potential to overcome the limitations of image sensors under rainfall conditions.

In the future, it would be necessary to further verify the difference in data for the speed group during rainfall, which was not clearly identified in this study, as well as the object detection performance based on color, which was not verified in this study. In addition, it is necessary to verify the performance of various LiDAR products in a real road environment for a comprehensive comparison. Continuous research and analysis of LiDAR performance in real road environments with various performance indicators will enable LiDAR to play a more active role in automated vehicles.

## Figures and Tables

**Table 1:** Test items used in the study for performance verification of LiDAR.

Changes of Environment on the Road	Performance Indicator	Theoretically Expected Results
Distance to the object target from vehicle (LiDAR)	NPCs	As the distance decreases, the NPCs gradually increase and then are maintained at a certain level.
	Intensity	As the distance increases, the intensity gradually increases.
Materials of the object target	NPCs	The NPCs are always maintained at a specific value.
	Intensity	The intensity is maintained at a specific value.
Driving speed of vehicle (LiDAR)	NPCs	The NPCs are always maintained at a specific value regardless of any change in speed.
	Intensity	The intensity is always maintained at a specific value regardless of any change in speed.
Rainfalls	NPCs	The NPCs decrease as rainfall increases.
	Intensity	The intensity decreases as rainfall increases.
Colors of the object target	NPCs	The NPCs decrease as the color of the target becomes more achromatic.
	Intensity	The intensity decreases as the color of the target becomes more achromatic.

**Table 2:** Technical Specifications of RS-LiDAR-32 (from product manual).

Sensor	Time of Flight Distance Measurement 32 Channels Measurement Range: 40 cm to 200 m (on 20% reflectivity target) Accuracy: ±3 cm Field of View: (Vertical) −25° to approximately +15°/(Horizontal) 360° Angular Resolution: (Vertical) at least 0.33°/(Horizontal) 0.1° to 0.4° Rotation Speed: 300/600/1200 rpm
Laser	Class 1 Wavelength: 905 nm Full Beam Divergence Horizontal: 7.4 mrad, Vertical: 1.4 mrad
Output	Data Rate: approximately 600,000 points/second 100 Mbps Ethernet UDP packet include: Distance, Rotation Angle/Azimuth, Calibrated Reflectivity, Synchronized Timestamp (Resolution: 1 μs)

**Table 3:** Classification of test scenarios.

Element	Item	Scenarios by Item					
Environmental Factor	Speed (Km/h)	80	60		40		20
	Distance (m)	100	80		60	40	20
	Rainfall (mm/h)	0 (Sunny Day)	10	20	30	40	50
Target Factor	Material	Wood	Plastic		Steel		Aluminum

**Table 4:** Result of the ANOVA test on whether the material of the target and the speed affect the NPC for each distance.

Hypothesis	Distance	Sunny Day
Hypothesis 1	100 m	Could not be analyzed
	80 m	Accepted
	60 m	Accepted
	40 m	Accepted
	20 m	Accepted
Hypothesis 2	100 m	Could not be analyzed
	80 m	Accepted
	60 m	Accepted
	40 m	Accepted
	20 m	Accepted

**Table 5:** Types of rainfall [26] used in the experiments.

Rainfall (mm/h)	Classification	Expressions
10 mm	Moderate Rain	The sound of raindrops falling on the roof of the vehicle is heard.
20 mm	Heavy Rain	Strong sound of rain. It becomes difficult to secure visibility without using the wipers.
30 mm		Heavy rainfall causes fields or sewers to start overflowing, with a high risk of rain damage. It is difficult to secure forward visibility even when the wiper is operated at normal speed.
40 mm		With the pouring rain at the level of heavy rainfall warning, it is difficult to secure forward visibility even when operating the wipers at its highest speed.
50 mm	Violent Rain	The vehicle should be driven at low speed even with wipers being operated at highest speed.

**Table 6:** Results of ANOVA test on the effect of the target material and speed on the NPC for each distance in the presence of rainfall.

Hypothesis	Distance	Rainfall 10 mm/h	Rainfall 20 mm/h	Rainfall 30 mm/h	Rainfall 40 mm/h	Rainfall 50 mm/h
Hypothesis 1	100 m	Rejected	Accepted *	Accepted	Rejected	Rejected
	80 m	Accepted	Accepted	Accepted	Rejected	Rejected
	60 m	Accepted	Accepted	Rejected	Rejected	Rejected
	40 m	Accepted	Accepted	Rejected	Rejected	Rejected
	20 m	Accepted	Accepted	Accepted	Accepted	Rejected
Hypothesis 2	100 m	Rejected	Accepted *	Accepted	Accepted	Accepted
	80 m	Accepted	Rejected	Accepted	Rejected	Accepted
	60 m	Accepted	Rejected	Accepted	Accepted	Accepted
	40 m	Rejected	Rejected	Rejected	Rejected	Rejected
	20 m	Rejected	Rejected	Rejected	Rejected	Rejected

* As the measured values were the same for Hypotheses 1 and 2 under the conditions of 100 m distance and 20 mm/h rainfall, ANOVA could not be performed, which could be interpreted as the absence of any difference according to material or speed.

**Table 7:** Result of ANOVA test on the effect of materials and speed on the intensity for each distance.

Hypothesis	Distance	Analysis Result
Hypothesis 1	100 m	Rejected
	80 m	Rejected
	60 m	Rejected
	40 m	Rejected
	20 m	Rejected
Hypothesis 2	100 m	Accepted
	80 m	Accepted
	60 m	Accepted
	40 m	Accepted
	20 m	Accepted

**Table 8:** Results of ANOVA test on the effect of target materials and speed on the intensity for each distance and for different amounts of rainfall.

Hypothesis	Distance	10 mm/h	20 mm/h	30 mm/h	40 mm/h	50 mm/h
Hypothesis 1	100 m	Rejected	Rejected	Rejected	-	-
	80 m	Rejected	Rejected	Rejected	-	-
	60 m	Rejected	Rejected	Rejected	Rejected	-
	40 m	Rejected	Rejected	Rejected	Rejected	Rejected
	20 m	Rejected	Rejected	Rejected	Rejected	Rejected
Hypothesis 2	100 m	Accepted	Accepted	Accepted	-	-
	80 m	Accepted	Accepted	Accepted	-	-
	60 m	Rejected	Accepted	Accepted	Rejected	-
	40 m	Rejected	Rejected	Accepted	Rejected	Accepted
	20 m	Rejected	Rejected	Accepted	Accepted	Rejected

**Table 9:** Summary of the performance validation results of the various test items for performance verification of LiDAR.

Changes of Environment on the Road	Performance Indicator	Theoretically Expected Results	Real Road Environment Analysis Results
Distance to the object target from vehicle (LiDAR)	NPC	As the distance decreases, the NPC gradually increases and then is maintained at a certain value.	Same as left
	Intensity	As the distance decreases, the intensity gradually increases.	Same as left (however, decreases at close range)
Materials of the object target	NPC	The NPC is maintained at a specific value according to target material.	NPC is measured uniformly regardless of the target material. However, in more than 40 mm/h of rain, the measured value depends on the material.
	Intensity	The intensity is maintained at a specific value according to target material.	Same as left
Driving speed of vehicle (LiDAR)	NPC	The NPC is always maintained at a specific value regardless of any change in speed.	Same as left
	Intensity	The intensity is always maintained at a specific value regardless of any change in speed.	Same as left
Rainfalls	NPC	NPC decreases as rainfall increases.	Same as left However, data loss begins to occur from 40 mm/h rainfall.
	Intensity	The intensity decreases as rainfall increases.	Same as left However, data loss begins to occur from 40 mm/h rainfall.

## Data Availability

Not applicable.
